# Research Laboratories for Mental Hospitals

**Published:** 1912-07-06

**Authors:** 


### The Hospital

The Workers' Newspaper of Administrative Medicine and Institutional Life, Administration, National Insurance and Health.

No. 1357, Vol. LII. SATURDAY, JULY 6, 1912. PRICE ONE PENNY.

### RESEARCH LABORATORIES FOR MENTAL HOSPITALS.

The treatment of mental disease in its various forms entails an enormous expenditure on the community. The actual monetary cost can be calculated, but there is a greater evil. Who that has studied the subject does not feel that our present knowledge and methods are not adequate to check the perpetuation, or even to stay the growth, of this incubus? Gigantic buildings, filled largely by the accumulations of incurable mental patients, stud the country. These might be styled monuments to the failure of the physician, were he alone to blame. The gibe would carry just enough truth to barb the dart were it not that physicians themselves are foremost in directing the attention of authorities to the question. The heads of our mental hospitals are calling out for facilities to pursue investigations into the origin of mental disease. They desire opportunities for increasing our knowledge of neuropathology, and for proving how far bio-chemistry may aid them in finding a cure for some forms of mental disorder. Skilled and well-directed efforts towards this end should be encouraged, if only on the low ground of being a good cash investment.

There is a higher ground which will be a more effective plea to nobler minds. The more enlightened laymen appreciate the position and are willing to assist. What stands in the way? The want of money. A large and wealthy body like the London County Council can found and equip a laboratory in connection with its mental hospitals. Under the able direction of Dr. Mott work has been done and is being done of which even the Metropolis may be proud. Good work on similar lines has been done in Edinburgh, Wakefield, and other places. Stimulated by the success already achieved and painfully aware of the mass of material which is not utilised, those interested in many of our mental hospitals throughout the country are anxious to follow so good an example. Endeavours are being made to associate various counties and county boroughs into districts for the purpose of establishing joint laboratories, which could then be equipped on a scale adequate and necessary for the work to be done. There is no intention to supersede such work as can be done at the separate institutions, but rather to foster and to supplement it. The director of the central laboratory would assist the local medical officers by advice. He would know what was being done and could systematise and make more perfect the investigations which were being carried out. A perfectly equipped laboratory and skilled assistance would be available for inquiries which were beyond the capabilities of the local medical officers or their appliances. We think that every five years the medical officers in our mental hospitals should have leave allowed for study. The exigencies of their work of necessity demand such opportunities for keeping up to date. This laboratory, especially if stationed in a town where there was a school of medicine, would be available for this purpose.

The suggested grouping of counties for so laudable an undertaking appears a natural and proper course. If the establishment and maintenance of a properly equipped and staffed laboratory be beyond the resources of the smaller authorities, why should they not unite in carrying out a scheme which under the London County Council has been productive of so much good? One is surprised to find that there are initial difficulties which, unless the Local Government Board intervene, may prove insuperable. The law sanctions the expenditure of public money by local authorities in order to provide facilities for laboratory work at each mental hospital, and allows them to pay the salary of a skilled pathologist. It is forbidden for authorities to unite in order to provide a laboratory more perfectly equipped and better managed than would be possible for a single authority to maintain. There can be no question under which scheme the better work would be done. A grant should be made by Parliament towards the erection of such establishments. We believe that few local authorities would decline to be associated with such inquiries. Any advance towards the prevention of insanity or its cure would repay the expenditure upon it. If a laboratory is properly to be maintained a sum of £1,000 a year will be necessary. It is poor economy to use cheap tools when important work is to be done. We foresee that the greatest difficulty lies not in the establishing of such institutions, but in their subsequent maintenance.

Public bodies change, and a council or committee cannot bind its successor. We have not so little faith in posterity or such conceit in our generation, however, as to suppose that the future will be less enlightened than the present, which complains of the heavy cost of our mental hospitals in the same breath as it protests against providing those laboratories, where alone, so far as our present and largely untried knowledge goes, resides a means of diminishing the number of cases.

